# Comparison of Lentiviral and Sleeping Beauty Mediated αβ T Cell Receptor Gene Transfer

**DOI:** 10.1371/journal.pone.0068201

**Published:** 2013-06-28

**Authors:** Anne-Christine Field, Conrad Vink, Richard Gabriel, Roua Al-Subki, Manfred Schmidt, Nicholas Goulden, Hans Stauss, Adrian Thrasher, Emma Morris, Waseem Qasim

**Affiliations:** 1 Molecular immunology Unit, Institute of Child Health, University College London, London, United Kingdom; 2 Department of Translational Oncology, National Center for Tumor Diseases and German Cancer Research Center, Heidelberg, Germany; 3 Institute of Immunity & Transplantation, Royal Free Campus University College London, London, United Kingdom; Chang Gung University, Taiwan

## Abstract

Transfer of tumour antigen-specific receptors to T cells requires efficient delivery and integration of transgenes, and currently most clinical studies are using gamma retroviral or lentiviral systems. Whilst important proof-of-principle data has been generated for both chimeric antigen receptors and αβ T cell receptors, the current platforms are costly, time-consuming and relatively inflexible. Alternative, more cost-effective, Sleeping Beauty transposon-based plasmid systems could offer a pathway to accelerated clinical testing of a more diverse repertoire of recombinant high affinity T cell receptors. Nucleofection of hyperactive SB100X transposase-mediated stable transposition of an optimised murine-human chimeric T cell receptor specific for Wilm’s tumour antigen from a Sleeping Beauty transposon plasmid. Whilst transfer efficiency was lower than that mediated by lentiviral transduction, cells could be readily enriched and expanded, and mediated effective target cells lysis *in vitro* and *in vivo*. Integration sites of transposed TCR genes in primary T cells were almost randomly distributed, contrasting the predilection of lentiviral vectors for transcriptionally active sites. The results support exploitation of the Sleeping Beauty plasmid based system as a flexible and adaptable platform for accelerated, early-phase assessment of T cell receptor gene therapies.

## Introduction

Gamma retroviral-mediated transfer of recombinant receptors to redirect T cell immunity against tumour and viral antigens is being extensively investigated in early phase clinical trials. Delivery of chimeric antigen receptors (CAR) against a variety of target antigens, including CD19 in haematological malignancies, GD2 in neuroblastoma and CEA in colonic carcinoma has been reported and reviewed recently [1]. Similar vectors have also been used to transfer conventional recombinant αβ T cell receptors against melanoma antigens [2]. Whilst there were concerns that these vectors may mediate genotoxic side effects in trials involving haematopoietic stem cell modification [3], [4], no vector related adverse events have been reported in T cell studies [5]. Recently, HIV-1 derived lentiviral vector based T cell redirection with a CAR against CD19 was reported [6], and these vectors are thought to improve efficiency of gene transfer and yield more functional T cells after *ex-vivo* transduction. Previously, both retroviral [7] and lentiviral [8] modification of T cells expressing recombinant T cell receptors against the Wilm’s tumour (WT1) antigen has been described and Phase 1 testing of an enhanced HLA-A2/WT1 peptide specific TCR has been initiated. However, high-costs associated with the manufacture of clinical grade viral vectors have been limiting, and to date only a handful of viral vectors, with limited target specificity have been produced. This limits applicability to individuals with specific HLA types and substitution of existing receptors with more efficacious derivatives is difficult. Alternatively, more readily adaptable gene delivery platforms, capable of mediating stable transgene integration without toxicity, could accelerate clinical testing and extend applicability. Plasmid-based gene transfer systems based on transposable elements are under investigation of a variety of gene therapy applications. The Sleeping Beauty (SB) platform [9] has been adapted to transfer antigen-specific receptors to T cells [10]–[14], but direct comparisons with lentiviral delivery are lacking. Here we have assessed the ability of an enhanced Sleeping Beauty transposition system, using a hyperactive SB transposase named SB100X [15] to deliver a codon optimised αβ TCR against WT1 antigen. This HLA-A2 restricted receptor has been modified to incorporate murine constant domains and additional disulphide bonds to mitigate against aberrant cross-pairing with endogenous TCR chains. Transduction efficiency, integration sites and redirection of human primary T cells against WT1 antigen was assessed and compared to lentiviral transduction procedures.

## Materials and Methods

### Ethics Statement

Informed written consent was obtained from all healthy blood sample donors with institutional ethics committee approval (University College London, UK). All animals were handled in strict accordance with good animal practice as defined by UK Home Office Animal Welfare Legislation, and all animal work was approved by the Institutional Research Ethics Committee (University College London, UK) and performed under Home Office approval (project license number 70/6482).

### Cell Lines and Primary T Cells

Human leukaemic immortalized Jurkat cell lines (TCR negative) [16] and TAP (transporter associated with Ag processing) deficient HLA-A0201^+^ T2 cell line [17] were maintained in RPMI-1640 (Invitrogen, Paisley, UK) supplemented with 10% heat-inactivated foetal calf serum (Sigma-Aldrich, Gillingham, UK), and 1% penicillin/streptomycin (Invitrogen). Human embryonic kidney 293T cells (ATCC CRL-11268™) were cultured in Dulbecco’s modified Eagle’s medium (Invitrogen) supplemented with 10% heat-inactivated foetal calf serum and 1% penicillin/streptomycin. Human peripheral blood mononuclear cells (PBMCs) were isolated from healthy HLA-A02 volunteers (with institutional ethics committee approval) by density gradient separation using Ficoll-Paque Plus (GE Healthcare, Little Chalfont, UK) and were cultured in X-VIVO 10 (Lonza, Cologne, Germany) supplemented with human AB serum (10%; Lonza) and the human recombinant common γ-chain cytokine IL2 (Chiron, France).

### Antibodies, Cytokines, Peptides and Flow Cytometry

Antibodies for flow cytometry were anti-human CD3 APC, CD8 FITC, anti-mouse TCRβ chain (mCβ APC) (BD Biosciences, Cowley, UK); anti-human Vβ2.1 PE and pWT126 tetramer PE (Beckman Coulter, High Wycombe, UK). The antibodies used for cytokine capture assay were IFNγ catch reagent and anti-human IFNγ PE (Miltenyi Biotech, Germany). The concentration of the human recombinant IL2 was 100 U/ml in all culture conditions. The peptides used in this study were the HLA-A0201-binding peptides pWT126 (RMFPNAPYL) and pWT235 (CMTWNQMNL), which were synthesized by ProImmune as described previously [18]. Flow cytometric analysis was performed using a LSR II flow cytometer (BDBiosciences) and the data were analysed using FlowJo version 7 software (TreeStar, Ashland, OR, USA).

### Lentiviral and Sleeping Beauty Constructs

A self-inactivating lentiviral vector, encoding the SFFV (spleen focus forming virus) LTR promoter and the HIV-1 central polypurine tract sequence and mutated Woodchuck post transcriptional regulatory element (WPRE) [19] was generated to include a codon-optimized hybrid HLA-A0201-restricted WT1-TCR genes [8]. The receptors comprise murine constant region with additional disulphide bonds and human variable (Vα1.5 and Vβ2.1) region sequences. The α and β chains are linked by a P2A self-cleaving peptide sequence and the TCR recognises WT126 peptide, RMFPNAPYL in the context of HLA-A0201 [8]. The SB transposon plasmid pT2/HB and hyperactive transposase plasmid pCMV-SB100X have been previously described [15], [20], [21] and were modified to encode an identical SFFV promoter WT1-TCR cassette.

### Lentiviral Vector Production

Lentiviral vector stocks were produced by transient co-transfection of subconfluent HEK293T cells using polyethylenimine (Sigma-Aldrich) with helper plasmids encoding vesicular stomatitis virus (VSV) envelope plasmid (pMDG), and gag/pol packaging plasmid (pCMVΔ8.74) as previously described [22]. Viral supernatant was harvested 48 and 72 hours later, 0.22 µM filtered, and concentrated by ultracentrifugation before cryopreservation. Vector stocks were titred on TCR negative Jurkat T cells by flow cytometry.

### Lentiviral Vector Transduction and Sleeping Beauty Nucleofection

PBMCs were activated using magnetic beads conjugated to antibodies to CD3 and CD28 (Invitrogen, Stockholm, Sweden), at a ratio of 1∶1 for 48 hours in X-VIVO 10 medium supplemented with human AB serum and 100 U/ml IL2 [22]. The cells were then exposed to lentiviral infection at a multiplicity of infection of 20 or nucleofected with DNA supercoiled plasmids (5 µg each) encoding the SB transposon and the SB transposase, using an Amaxa Nucleofector II device and Human T Cell Nucleofector Kit and protocol for Stimulated Human T cells (Lonza). After 48–72 hours after expression of TCR transgenes was analysed by flow cytometry.

### Expansion and Purification of TCR-modified T Cells

TCR modified T cells were either assessed immediately or expanded weekly by antigen-specific stimulation in 24-well plates as follows: 5×10^5^ TCR-transduced cells were co-cultured with 2×10^5^ T2 cells pulsed with pWT126 peptide as stimulators and 2×10^6^ irradiated autologous feeder PBMCs, in the presence of 100 U/ml IL2. In some experiments, WT1-TCR T cells were expanded and then enriched by sorted by magnetic bead selection using MiniMacs columns following two stage incubation with an anti-murine TCRβ chain APC antibody, followed by anti-APC microbeads (Miltenyi Biotech).

### Cytotoxicity Assays

In vitro ^51^Cr release cytotoxicty assays were performed with 2×10^6^ T2 cells pre-incubated at 37°C for 1 hour in 200 µl assay medium (RPMI 1640 containing 5% heat-inactivated FCS) with 100 µM specific peptide as target cells. Peptide-coated T2 cells were then labelled with ^51^Cr for 1 hour, washed, and added to serial 2-fold dilutions of expanded WT1-TCR modified PBMCs as effector cells in round-bottom 96-well plates to obtain a total volume of 200 µl/well. Assay plates were incubated at 37°C, 5% CO2, and after 4 hours, 50 µl supernatant was harvested and radioactivity counted. The specific killing was calculated by the equation: (experimental release-spontaneous release)/(maximum release-spontaneous release) ×100%.

In vivo cytotoxicity assays were performed with 0.7×10^6^ WT1-TCR modified splenocytes from C57Bl/6 mice as effector cells, which were activated with Concanavalin A 2 µg/ml (Sigma-Aldrich) and IL2 100 U/ml for 3 days in RPMI 1640 containing 10% heat-inactivated FCS, 0.1 mM 2-mercaptoethanol (Invitrogen) and 0.1 mM sodium pyruvate (Lonza) and nucleofected with the SB transposon and the SB transposase DNA plasmids, using an Amaxa Nucleofector II device and Mouse T Cell Nucleofector Kit and protocol (Lonza). The effector cells were injected intravenously into A2Kb mice without conditioning and left 2 days to equilibrate. Splenocytes from female A2Kb Tg mice were prepared as target cells by selecting B cells (B Cell Isolation Kit; Miltenyi Biotec), peptide-loaded with 100 µM of either specific peptide (WT126p) or an irrelevant HLA-A0201-presented peptide before labelling with 1.5 µM or 0.15 µM CFSE, respectively, for 5 minutes at 37°C. Labelled cells were mixed at a 1∶1 ratio, relevant: irrelevant targets and a total of 10×10^6^ mixed cells were injected into recipient A2K^b^ mice previously injected with effector cells. Eighteen hours later, splenocytes of injected animals were analyzed by flow cytometry to identify CFSE-labelled cells. Control untreated A2K^b^ Tg mice were injected with labelled target cells only and in vivo cytotoxicity was calculated as previously described. Percentage antigen-specific cytotoxicity was determined using the following formula: [1- (mean number of relevant peptide-loaded targets in experimental mice/mean number of irrelevant peptide-loaded targets in experimental mice)/(mean number of relevant peptide-loaded targets in control mice/mean number of irrelevant peptide-loaded targets in control mice )] ×100.

C57Bl/6 mice were purchased from Charles River (Margate, UK). HLA-A0201K^b^ transgenic (A2K^b^ Tg) mice on a C57Bl/6 background were a kind gift from Theobald M. (University Medical Center, Utrecht, The Netherlands) and were bred and maintained in the Comparative Biology Unit of University College London (Royal Free Hospital, London, UK) under pathogen-free conditions.

### Ligation-mediated PCR (LM-PCR)

To recover integration sites, DNA from WT1-TCR modified PBMCs by lentiviral transduction or SB nucleofection was extracted by Proteinase K digestion. 1 µg of DNA was digested with NlaIII to produce vector-chromosome junction fragments, ligated to excess linker and PCR was performed with GoTaq polymerase (Promega, Madison, WI) using the linker first-round and second-round primers as previously described [20], [21]. PCR products were shotgun-cloned into the Topo-TA vector (Invitrogen) and sequenced (UCL, London, UK) using the second round primers. LM-PCR products were 454 pyrosequenced as previously described [21].Raw sequence reads obtained after sequencing were trimmed and aligned to the human genome (NCBI, hg19) using HISAP [23]. Integration sites were considered to be valid if vector-genome junction sequence was present and the flanking genomic region had a unique sequence match of at least 95% after alignment to the human genome (University of California at Santa Cruz, RefSeq genes and RepeatMasker). Chromosome graphs were generated using the University of California at Santa Cruz Genome Graphs tool.

### Statistics

Where shown, error bars represent the standard error of the mean of the replicates indicated, and differences were found to be significant by application of non-parametric Anova tests.

## Results

### 1. Sleeping Beauty Mediated αβ TCR Transfer to Primary T Cells Compared to Lentiviral Delivery

**Figure 1:**
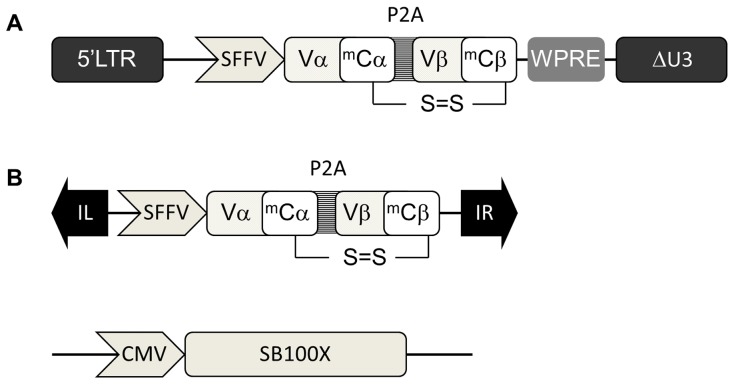
WT1-TCR vector constructs. A. Schematic representation of the pSIN second-generation lentiviral (Lv) vector encoding the codon-optimized, murinized hybrid HLA-A0201–restricted pWT126-specific additional cysteines modified TCR α- and β-chain genes separated by a self-cleaving porcine teschovirus 2A sequence (P2A). LTR indicates long terminal repeat; m, murine SFFV, spleen-forming focus virus; and WPRE, woodchuck hepatitis virus posttranscriptional regulatory element. B. Schematic representation of the plasmid Sleeping Beauty (SB) vector consisting of a transgene expression cassette, encoding the codon-optimized, murinized hybrid HLA-A0201–restricted pWT126-specific additional cysteines modified TCR α- and β-chain genes separated by a self-cleaving porcine teschovirus 2A sequence (P2A), flanked by inverted repeats; and the hyperactive SB transposase SB100X. CMV indicates cytomegalovirus promoter; IL, left inverted repeat; IR, right inverted repeat; m, murine; SFFV, spleen-forming focus virus.

Sleeping Beauty transposon-mediated TCR delivery was compared against lentiviral vector-mediated gene transfer in primary human lymphocytes using identical promoter/transgene expression cassettes. These comprised an optimised human-murine chimeric WT1 αβTCR encoding Vα1.5 and Vβ2.1 chains [8] in combination with the spleen-forming focus virus (SFFV) long terminal repeat promoter (Figure 1A, B). Sleeping Beauty transposons were delivered in combination with a functionally enhanced, hyperactive, Sleeping Beauty transposase variant, SB100× (Figure 1B). Preliminary experiments in TCR-deficient Jurkat T cells allowed optimisation of transposon/SB100X plasmid ratios, and confirmed robust and durable expression following both lentiviral and SB delivery.

**Figure 2:**
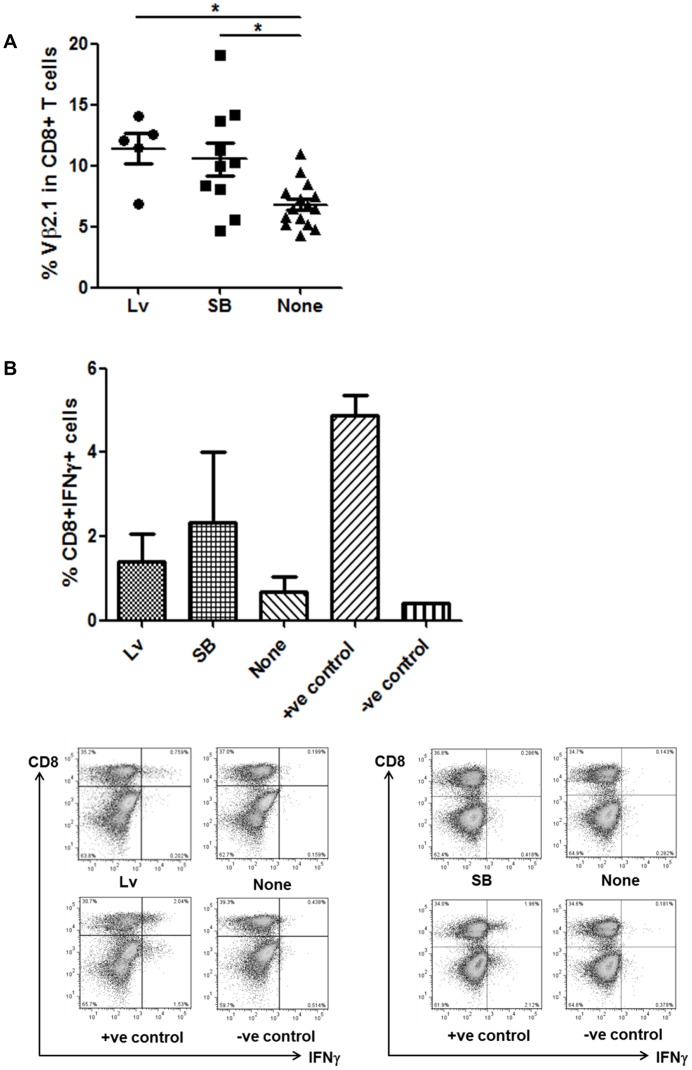
WT1-TCR expression and function in human primary T cells. A. WT1-TCR cell surface expression. Percentages of Vβ2.1 positive cells in CD8+ T cells for each HLA-A2+ donor are shown after WT1-TCR lentiviral (Lv) or Sleeping Beauty (SB) vector gene transfers. CD3/CD28 microbeads-activated PBMCs were transduced at a multiplicity of infection of 20 or nucleofected with 5 µgs transposon and transposase plasmids. *p<0.05. B. Antigen-specific effector function. Mean percentages of IFNγ positive cells in CD8+ T cells from HLA-A2+ donors assayed by MACS Cytokine Secretion Assay of human primary CD8+ T cells, after WT1-TCR lentiviral (Lv) or Sleeping Beauty (SB) vector gene transfer, restimulated with either the specific WT1 peptide, anti-CD3 Ab (positive control) or irrelevant peptide (negative control), and autologous PBMCs (top). Representative dot plots of IFNγ secretion are also shown after WT1-TCR Lv (bottom left) or SB (bottom right) gene transfer.

**Figure 3:**
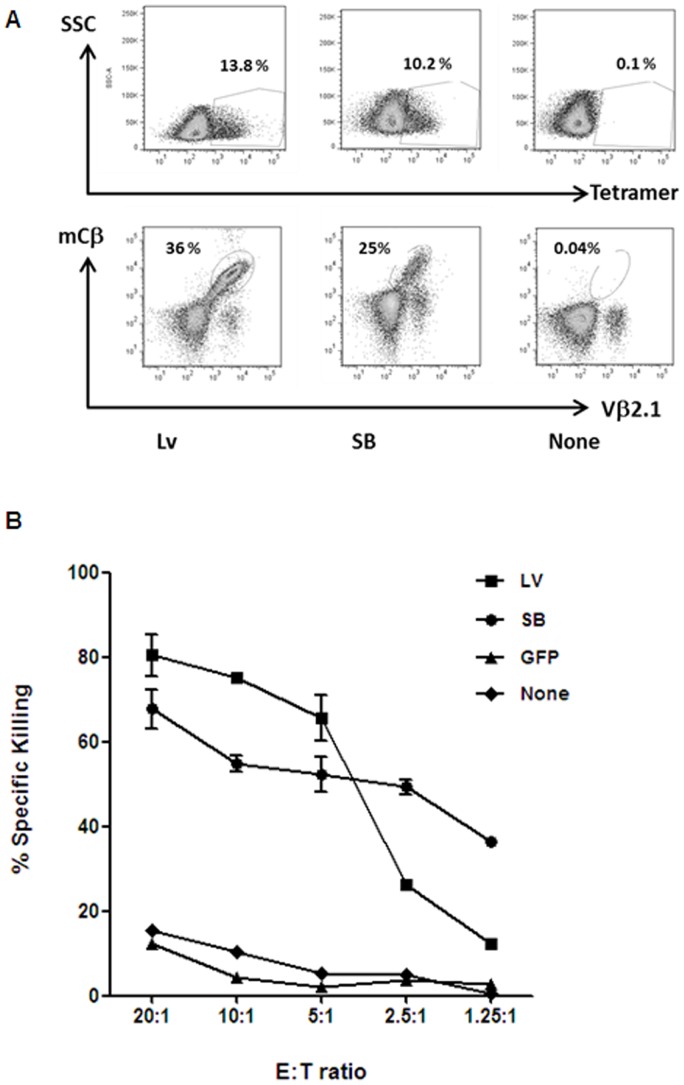
Antigen-specific responses of human primary T cells. A. Murine Cβ/human Vβ2.1 expression and WT1 tetramer binding are shown in representative human primary lymphocyte dot plots, after SFFV-driven WT1-TCR lentiviral vector (Lv) or Sleeping Beauty (SB) gene transfer and in unmodified controls. CD3/CD28 microbeads-activated PBMCs were transduced at a multiplicity of infection of 20 or nucleofected with 5 µgs transposon and transposase plasmids and expanded with T2 cells coated with specific peptide pWT126 and irradiated autologous PBMCs as feeder cells. B. *In vitro* cytotoxicity of donor T cells against specific peptide-loaded T2 target cells, Lv versus SB. Percentages of cytolytic activity of T cells, after WT1-TCR lentiviral (Lv) or Sleeping Beauty (SB) vector gene transfers, was tested in a ^51^Cr release CTL assay. CD3/CD28 microbeads-activated PBMCs were transduced at a multiplicity of infection of 20 or nucleofected with 5 µgs transposon and transposase plasmids and expanded with the specific WT1 peptide and autologous irradiated PBMCs. Both WT1-TCR-modified donor T cells recognized and killed their specific targets (2 way ANOVA, p<0.001 at all E:T ratios compared to GFP-modified or unmodified cells).

Freshly isolated human peripheral blood lymphocytes (PBL) from healthy HLA-A2 donors were stimulated for 48 hours with anti-CD3/CD28 microbeads and human IL-2 then exposed to a single round of lentivirus infection or nucleofected with transposon/transposase DNA plasmids. Flow cytometry following staining with anti-Vβ2.1 antibody identified primary T cells expressing cell surface WT1-TCR and revealed comparable gene transfer. These investigations using multiple healthy volunteer donor lymphocytes revealed inter-individual variability, with mean levels of SB mediated gene transfer in CD8^+^ T cells of 10.5% (n = 10) compared to 11.4% (n = 5) following lentiviral transduction (Figure 2A). These engineered CD8^+^ T cells were pulsed with HLA-A2 restricted WT1 peptide and antigen-specific responses were confirmed on the basis of IFNγ release (Figure 2B). Assuming that WT1 specific responses were only mediated by WT1-TCR expressing cells, events captured by flow cytometry within the CD8+IFNγ+ gate (1.4% for LV, 2.3% for SB) accounting for 28% and 57% of the modified populations respectively. Furthermore, WT1-TCR-modified T cell populations could be expanded and were then subjected to challenge by peptide loaded target cells. Co-staining for murine TCRβ chain epitopes distinguished recombinant receptor expression from endogenous Vβ2.1 expression and HLA-A2/pWT126 multimer staining confirmed WT1-specific TCR expression (Figure 3A). Human TAP-deficient T2 target cells loaded with pWT126 peptide were specifically lysed by both lentiviral and SB modified T cells, in contrast to cells presenting non-relevant peptides or cultures challenging control (eGFP) modified T cells (Figure 3B).

### 2. *In vivo* Functional Assessment of SB Modified T Cells

**Figure 4:**
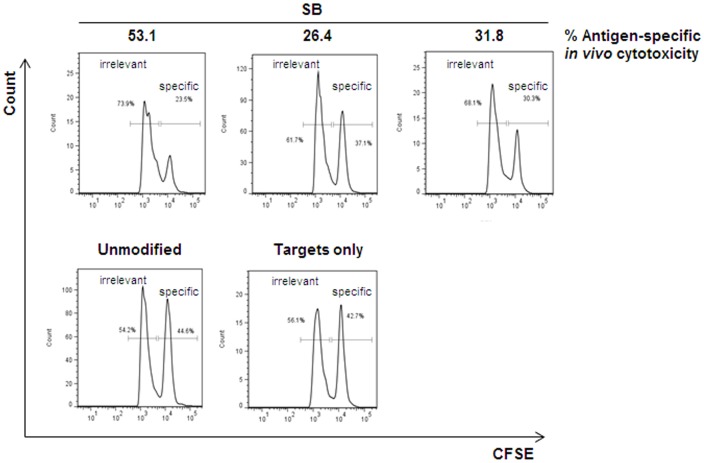
*In vivo* cytotoxicity of murine primary T cells. *In vivo* cytotoxicity of modified mouse splenocytes against CFSE-labelled peptide-loaded mouse target B cells. A2K^b^ Tg mice, 2 days after intravenous injection of syngeneic effector cells modified with Sleeping Beauty (SB) WT1-TCR vector system, were again intravenously injected with a 1∶1 mix of relevant: irrelevant peptide-loaded A2K^b^ Tg target B cells, differentially labelled with CFSE (specific WT126 peptide, 1.5 µM CFSE, and irrelevant WT235 peptide for WT1-TCR, 0.15 µM CFSE). Eighteen hours later, splenocytes of injected animals were harvested and analysed by FACS to identify CFSE-labelled cells. Control A2K^b^ Tg mice were injected with only CFSE-labelled peptide-loaded target B cells or with unmodified splenocytes as effectors. Antigen-specific cytotoxicity was calculated as [1- (number of relevant peptide-loaded targets in experimental mice/number of irrelevant peptide-loaded targets in experimental mice)/(number of relevant peptide-loaded targets in control mice/number of irrelevant peptide-loaded targets in control mice )] ×100.

Assessments of TCR redirected human T cells in immunodeficient mice injected with human WT1 expressing tumour cells have previously demonstrated the potency of retroviral modified T cells in vivo, including T cell homing and tumour eradication. However, these models are technically challenging with variable reproducibility. Alternatively A2K^b^ transgenic mice have been used to assess the function of T cells derived from murine stem cells modified using lentiviral vectors to express HLA-A2/WT1 specific TCRs [24]. Mature murine T cells resist lentiviral transduction through the expression of innate restriction factors [25] but we successfully adopted the model for confirmation that SB modified murine T cells expressing human TCR specific for HLA-A2/WT1 are functional in vivo. Activated splenocytes from C57Bl/6 mice were nucleofected with the SB transposon system expressing WT1-TCR and were injected into unconditioned recipient A2K^b^ transgenic mice. Two days later, target B cells from female A2K^b^ Tg mice were peptide-loaded with relevant (pWT126) or irrelevant HLA-A2-restricted peptides and labelled with CFSE. Labelled target with relevant or non-relevant targets were mixed at a 1∶1 ratio and were injected in the same recipient mice. Eighteen hours later, splenocytes of injected animals were analysed by flow cytometry to identify CFSE-labelled target cells. Control (untreated) A2K^b^ Tg mice were injected with only labelled target cells (Figure 4). In vivo, target specific cytotoxicity ranged from 26–53%, confirming antigen-specific function of SB modified T cells.

### 3. Sleeping Beauty Transposition Sites in Primary Human T Cells

**Figure 5:**
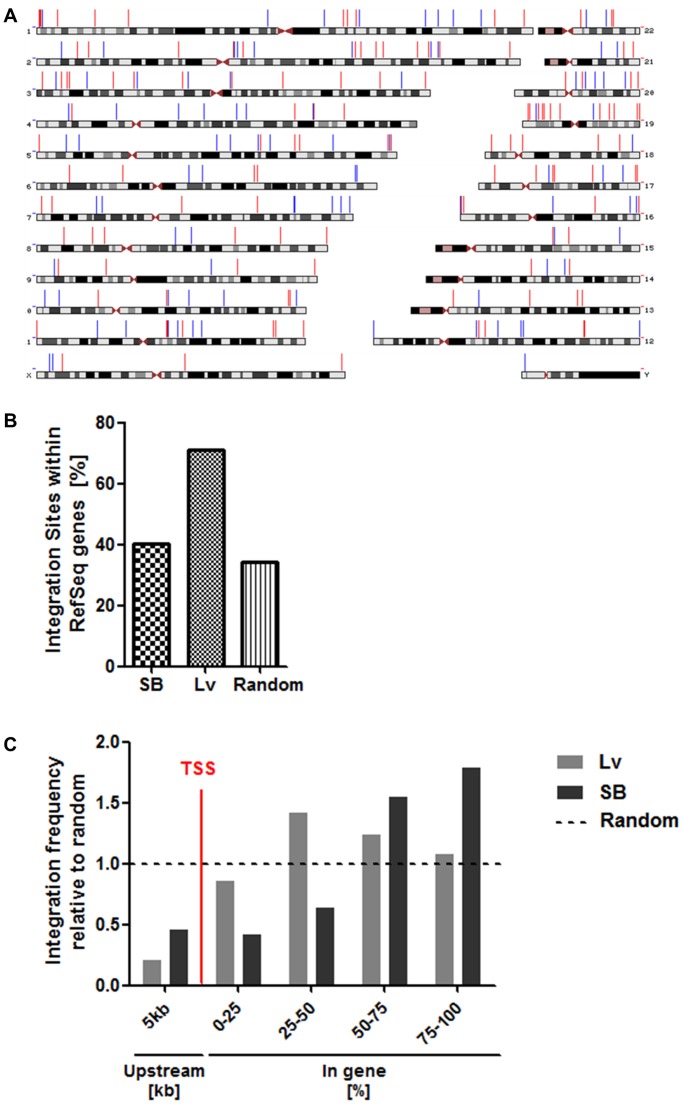
Integration profiles in human primary T cells by LM-PCR. CD3/CD28 microbeads-activated PBMCs were transduced at a multiplicity of infection of 20 (Lv) or nucleofected with both 5 µgs transposon and transposase plasmids (SB). Both vectors encoded the identical WT1-TCR transgene. Genomic DNA was extracted and lentivirus-chromosome or transposon-chromosome junctions were recovered by ligation-mediated PCR and sequenced. A. Genome-wide mapping of vector integrations at the chromosome level. Sequences were mapped to the University of California at Santa Cruz (UCSC) human genome by BLAT search and integration sites were depicted relative to chromosomes using the UCSC Genome Graphs tool. Lv in Blue, SB in Red. B. Frequency of integration sites of vectors within RefSeq genes. 1,000 random integration sites were generated by bioinformatics, as already described [21]. C. Proximity of integration sites to transcription start sites (TSS). RefSeq genes containing integration sites were divided by length into 4 equally sized regions and 1 upstream region (0–5 kb), and the proportion of integration sites within each region was counted. To allow statistical comparison of integration preferences with average genomic content, 1,000 random chromosomal sites were generated by multiplying the total length of the genome by a random number between 0 and 1 and converting this value to a chromosomal coordinate. Vector integration frequencies are expressed relative to the proportion of random sites within each region.

Lentiviral integration sites in T cells have been well characterised in subjects with HIV-1 infection and in gene engineered T cells and there is a preference for integration in transcriptionally active regions, though in contrast to gamma-retroviral vectors, no bias towards transcription start sites (TSS). Sleeping Beauty transposons target TA dinucleotides and are thought not to target gene coding sequences. Integration sites (n≥150) of lentiviral vectors and SB transposons expressing WT1-TCR transgenes were detected by high throughput sequencing. Overall, 70.8% of lentiviral integrations were found within genes compared to 40.2% of SB100X/transposon-mediated transgene insertions, which was more comparable to anticipated frequencies of 34.2% if random integration had occurred (Figure 5B). When integrations within genes were mapped relative to their position within the gene or upstream region (Figure 5C), no preference toward TSS of RefSeq genes was observed along the length of the gene and no significant variation in integration pattern was observed along the length of the gene. The bias towards RefSeq genes can be expressed as a ratio of frequencies in genes above random, and this yielded values of 2.05 for lentiviral vectors and 1.17 for the SB platform. Thus, in primary human lymphocytes plasmid-based SB delivery of αβTCR gene expression cassettes appears less likely to perturb endogenous gene function.

## Discussion

Wilms’ Tumour antigen 1 (WT1) is a well characterised tumour antigen overexpressed by haematological and certain non-haematological malignancies. A phase 1 study of gamma retrovirally modified T cells to treat relapsed leukaemia has recently been initiated to investigate the safety and efficacy of a recombinant αβ TCR specific for HLA-A2/WT1 peptide antigen (ClinicalTrials.gov; NCT01621724). As with other T cell modification therapies requiring stable transgene integration, a major hurdle has been the production and validation of clinical grade vector stocks. In the case of gamma-retroviral vectors, efficient stable packaging cell lines have facilitated the production of large volumes of vector stocks, and yet the very high levels of investment required have meant only a handful of candidate antigen-specific receptors have been adopted for translational studies. Recent advances support a switch to lentiviral vector platforms [22], [26], but GMP production of high titre lentiviral stocks is problematic and is hampered by the lack of stable producer lines. Rather, lentiviral stocks are produced by transient transfection, using high quality plasmids and then require extensive and costly downstream validation and release testing. The availability of an alternative plasmid based system for direct modification of T cells would allow accelerated assessment of different TCRs and revised CAR configurations. In this regard transposon-based systems are attractive and proposals for initial clinical testing of a nucleofection-based protocol for transfer of a CD19 specific CAR are well developed [14], [27], [28]. The enhanced SB100X mutant has been evaluated for transfer of a similar single chain receptor, but information on delivery of a αβ TCR configuration has been lacking. We have demonstrated stable gene transfer by SB100X-mediated transposition, and have determined that integration sites in primary human T cells are similar to those reported for human cell lines, and are not skewed towards gene rich, transcriptionally active loci. We speculate that this may reduce the risk of genotoxicity, albeit noting that there have been no vectors-mediated adverse events in any gamma retroviral or lentiviral studies targeting human T cells even when integrants close to protoncogenes have been detected. We speculate that this relates to the differentiated nature of T cells, in contrast to haematopoietic stem cells where risks of transformation may be greater. There have been concerns that background integration of transposase encoding plasmid may mediate remobilisation of transposed sequences, and this could also be a risk factor for mutagenesis. Strategies to mitigate against such eventualities include the use of mRNA rather than plasmid for transposase delivery [11], or the incorporation of a linked suicide gene for elimination of cells in case of adverse effects [22]. Whilst transposition may be less efficient than lentiviral-mediated gene transfer, we have demonstrated the feasibility of enriching cells by magnetic bead selection and expanding antigen-specific populations using stimulator cells and feeder layers if high numbers of cells are required [14], [29]. Alternatively, it is entirely possible that the early reinfusion of fewer SB modified T cells, cultured for a shorter period and retaining functional properties may be sufficient to mediate beneficial in vivo effects.

In summary, SB-mediated transfer of antigen-specific T cell receptor redirects effector function and offers a flexible pathway for rapid evaluation of different receptor configurations. Further optimisation and adaptation to GMP conditions will allow therapeutic applications to be explored in early phase clinical studies.
